# Life Course Effects of Socioeconomic and Lifestyle Factors on Metabolic Syndrome and 10-Year Risk of Cardiovascular Disease: A Longitudinal Study in Taiwan Adults

**DOI:** 10.3390/ijerph15102178

**Published:** 2018-10-05

**Authors:** Chen-Mao Liao, Chih-Ming Lin

**Affiliations:** 1 Department of Applied Statistics and Information Science, Ming Chuan University, Taoyuan 333, Taiwan; cmliao@mail.mcu.edu.tw; 2 Department of Healthcare Information and Management, Ming Chuan University, No. 5, Teh-Ming Rd., Gwei-Shan, Taoyuan 333, Taiwan

**Keywords:** metabolic syndrome, cardiovascular disease, socioeconomic status, lifestyle, life course

## Abstract

The objective of the study was to explore the dynamic effects of socioeconomic status (SES) and lifestyle behaviors on the risks of metabolic syndrome (MS) or cardiovascular disease (CVD) in life course. The data of 12,825 subjects (6616 males and 6209 females) who underwent repeated examinations and answered repeated questionnaires from 2006 to 2014 at the Major Health Screening Center in Taiwan, was collected and analyzed. The trajectory of trends in the subjects’ SES and lifestyle mobility over time was observed, and the effects of factors with potential impacts on health were tested and analyzed using multiple logistic regression and a generalized estimated equation model. A 10% increase in MS prevalence was observed over the nine-year period. The average Framingham CVD score for people with MS was estimated to be about 1.4% (SD = 1.5%). Except for middle-aged women, marriage was found to raise the risk of CVD, whereas increasing education and work promotions independently reduced CVD risk for the majority of subjects. However, the risk of CVD was raised by half for young men who had a job or lost a job in comparison to continuously unemployed young men. Physical activity was only found to be advantageous for disease prevention in those aged less than 40 years; increased exercise levels were useless for reducing CVD risk among older men. Alcohol drinking and betel chewing caused increased CVD risk in the old and young subjects, respectively, whereas vegetarian diets and vitamin C/E intake were helpful in preventing CVD, even if those habits were ceased in later life. For middle-aged women, getting sufficient sleep reduced CVD risk. We concluded that SES and lifestyle behaviors may have different effects on health over time, among various populations. Accordingly, suggestions can be provided to healthcare workers in designing health promotion courses for people at different life stages.

## 1. Introduction

Metabolic syndrome (MS) is characterized by multiple cardiovascular risk factors and causes health problems for individuals, as well as burdens on health care systems. The prevalence of MS appears to be rising due to increasing obesity rates and aging populations [1,2,3,4,5,6,7,8,9]. The potential factors underlying MS have been linked with demographic characteristics and lifestyle behaviors, such as smoking, drinking, dietary behaviors, and physical activity [10,11,12,13]. Antioxidants (e.g., vitamin C and vitamin E) have also been reported to be associated with a reduced risk of MS. Relatedly, a deficiency in plasma vitamin C is associated with reduced fat oxidation during aerobic exercise [13,14,15,16]. Moreover, socioeconomic status (SES) may have a strong association with cardiovascular disease (CVD) and poor metabolic indicators, whilst associations between MS and income, occupation, and education have been observed in several cross-sectional studies [17,18,19,20]. Nonetheless, a Korean study reported that the causal relationship between SES and both risk of MS and Framingham risk score (FRS) is unable to be established with a cross-sectional study [21]. 

It is therefore expected that exposure to socioeconomic or behavioral adversities during life also increases an individual’s long-term cardiovascular risk. The accumulation of risk model advocates that increasing the number, duration, and severity of adverse events over the life course increases the risk of disease development [22,23]. The concept of social mobility refers to the degree of SES stability or change in the trajectory of an individual’s life course itself [24]. Hoffmann et al. suggested that a life course approach can contribute to a better understanding of how a reciprocal relationship between affected factors and health changes over different life stages [25]. A study conducted in the USA found that the risk factors of CVD trajectories may be determined early in life, and that the effects of SES on risk may be long lasting, even as upward SES mobility may lead to some reduction of risk in young adults [26]. Using different SES indicators over different life stages, a recent Brazilian study reported that a life course with persistent low SES may also affect the 10-year CVD risk via the accumulation of risk and lack of social mobility [27]. However, this effect may differ across genders. Meanwhile, an earlier longitudinal study from Taiwan, which utilized data from health check-ups, found a 12.5% 5-year cumulative incidence of MS and a raised occurrence of MS in women with low education as they became older, in addition to finding that SES exhibited a greater influence on women than on men, in general [28]. 

Nonetheless, there is a lack of evidence as to whether the adverse SES and lifestyle behaviors simultaneously increase the risk of MS or CVD over time, as well as to whether any such increases to risk can be reversed when adverse conditions are improved upon in later life stages. Therefore, the current study used data from a large database of health check-up data to investigate whether exposure to changes in SES or lifestyle habits over the course of life are associated with MS or the 10-year risk of CVD.

## 2. Materials and Methods

### 2.1. Data Source

The study collected and analyzed data from the Major Health Screening Center in Taiwan. The center is a membership-oriented private institute with four clinics located around the country. These clinics provide periodic health examinations to the center’s approximately 610,000 members. Each member participated in a check-up program that offers a discounted examination fee for coming back to receive the examination repeatedly over multiple years. The data collection and analysis of the resulting Major Longitudinal Health-check-up-based Population Database (MJLPD) has been described in detail elsewhere in References [28,29]. The MJLPD database is made accessible to academic researchers upon request. Owing to usage of the data therein, entailing various ethical issues, the protocol of this study was evaluated and agreed to by the Research Ethics Committee, National Taiwan University (NTU-REC 201612ES009) and the Major Health Screening Center.

### 2.2. Study Sample

We conducted a longitudinal study of participants who underwent at least one standard health screening at the Center in each of three three-year stages/periods (i.e., 2006–2008, 2009–2011, and 2012–2014), from 2006 to 2014. All the participants with any missing questionnaire or examination data were excluded from the study. A final total of 12,825 subjects (6616 males and 6209 females) met the inclusion criteria for analysis. For those subjects who underwent multiple screenings in any three-year period, we selected the last examination for our analysis. Therefore, three questionnaires and examination measurements were collected for each participant during the overall nine-year period. The average individual follow-up duration was 5.45 years (with a standard deviation of 0.76 years). The intervals between any two measurements ranged from one year to three years.

### 2.3. Response Variables

In our study, MS was defined according to the modified ATP III criteria [30] and the official criteria announced by National Health Promotion Administration in Taiwan. The diagnosis of MS was made when 3 or more of the following were present: Waist circumference ≥ 90 cm in men and ≥80 cm in women; fasting glucose ≥ 100 mg/dL (5.55 mmol/L) or use of antidiabetic medication; systolic blood pressure (BP) ≥ 130 mmHg, diastolic BP ≥ 85 mmHg, or use of antihypertensive medication; fasting triglycerides ≥ 150 mg/dL; and high-density lipoprotein cholesterol <40 mg/dL in men and <50 mg/dL in women. To study the effects of MS on health over time, we created the following three-category indicator of ongoing MS status for the three stages, for those without (or with) MS initially: Stable health, worse (or improved) health, and unstable health. Worse health was defined as the occurrence of MS in the second and/or third stage, for those without MS in the first stage. In contrast, improved health was defined as recovery from MS over time, for those with MS initially. Individual 10-year CVD risk was estimated by the FRS applied in a Chinese population [31], which was treated as a continuous variable in this longitudinal study.

### 2.4. Explanatory Variables

The study subjects had each completed a self-administered questionnaire during screening to provide information on their sociodemographic characteristics and lifestyle habits. In addition to sex and age (classified into 20–39 years, 40–64 years, and 65 or more years), we collected data regarding four aspects of SES (i.e., marriage, education, income, and occupation) and nine lifestyle habits including smoking, drinking alcohol, betel nut chewing, physical activity, sleep, vegetarian diet, drinking sweetened beverages, and taking nutritional supplements (i.e., vitamin C/E or fish oil), which are well-documented as constituting related risk factors in past studies. Treatment with medicines such as antihyperglycemic, antihyperlipidemic, or antihypertensive drugs was used to identify MS, which also played the role of confounder in the multi-variable analysis.

Body mass index (BMI, kg/m^2^) was considered a continuous variable, and different BMI levels were classified according to the definition by the Ministry of Health and Welfare, Taiwan. Neglecting the BMI for underweight individuals (BMI < 18.5), which is not considered a risk factor for MS, three levels of BMI (specifically, <24, 24–6.9 (overweight), and ≥ 27 (obesity)) were used in our analysis.

With regard to SES, the participants were classified into three levels of education attainment: <12 years (lower than high school), 12–15 years, and > 15 years (higher than high school). Individual annual income was divided into three levels: Less than 400,000, 400,000 to 800,000, and > 800,000 (in New Taiwan dollars, NTD). Occupation was classified as unemployed (including students, housekeepers, the retired, etc.), non-management employee, and manager/owner. Married status was classified as married or unmarried. Some lifestyle habits which can be estimated quantitatively by frequency were classified into three levels: None, low (1–6 pack/week), and high (≥7 pack/week) for smoking; none, low (1–6 cup/week), and high (≥7 cup/week) for alcohol consumption; none, low (1–6 cup/week), and high (≥7 cup/week) for sweetened beverage consumption; low (<1 h/week), medium (1–6 h/week), and high (>6 h/week) for physical activity (PA); and low (<6 h/day), medium (6 h/day), and high (>6 h/day) for sleep, respectively. The other habits such as betel nut chewing, vegetarian diet, and taking nutritional supplements were classified dichotomously.

The trajectory of socioeconomic and lifestyle mobility was observed over the course of time. The categories were grouped by measuring the level across the three study stages as follows: Stable, none, or low level in three stages, increasing (i.e., rising level in the second and/or third stage), declining (decreasing level in the second and/or third stage), stable medium/high level across the three stages, and unstable condition. The dichotomous variables were classified as increasing when they occurred in a subsequent stage after being absent initially, whereas they were classified as decreasing when they did not occur in a subsequent stage after being present initially. An individual’s level of education accumulates over the life course; thus, there were no declines in this explanatory variable.

### 2.5. Statistical Analyses

The demographic, socioeconomic, and lifestyle characteristics, as well as the physical and biochemical aspects of the study subjects, with or without MS, over the three study stages were first described. Next, we assessed the dynamic associations between MS and demographic, socioeconomic, and lifestyle characteristics with multivariate logistic regression methods. The two groups classified as with or without MS in the first stage, were analyzed separately. Using the continuous status of MS or without MS in the three stages as the baseline, the impacts on improved/worse or unstable health status of MS progression could then be assessed, respectively. Using the stable, none, or low level in the three stages as the baseline, the same approach was also used to identify the trends over time (i.e., increase, decline, or unstable condition), for the explanatory variables. In addition, a promotion in terms of occupation might consist either of getting a job or taking a management position, after being unemployed or in a non-management position in the previous stage. Meanwhile, a subject who got divorced or became a widow after being married in the previous stage was defined as changing to being single, whereas those who got married after being single were defined as changing to being married. The adjusted odds ratios (AOR) and their 95% confidence interval (CI) were estimated using multiple logistic regression. Furthermore, the assessments were stratified according to sex and age, because they were considered possible interaction factors with the other explanatory factors. To observe the longitudinal association of continuous FRS and the potential factors, linear regression models were also fitted in generalized estimated equations (GEE) with a log link function and AR(1) structure. All analyses were adjusted to avoid confounding with individual CVD related medicine treatments. R version 3.2.5 (R Foundation for Statistical Computing, Vienna, Austria. 2016) was used for the aggregation of the data. Dichotomy analyses and the GEE model were performed with SAS version 9.1 (SAS Institute Inc., Cary, NC, USA).

## 3. Results

### 3.1. Descriptive Statistics

The results of the sociodemographic factors, lifestyle habits, and physical and biochemical examination items, through the three study stages, are shown in Table 1 and Table 2. The prevalence of MS in men and women at the first stage was 31.7% and 12.8%, respectively. An increase in MS prevalence (of approximately 10%) was observed in the following stages. The prevalence of MS for those who were obese (BMI ≥ 27), increased from 67.2% at the first measurement to 76.5% at the last. The average Framingham CVD score was estimated to be about 1.4% (±1.5%), for a person with MS.

### 3.2. Association of SES and Lifestyle Habits with Risk of MS or CVD

Table 3 shows the effects of changes in status for related factors, on a person with or without MS over time. The results indicated that men had a two- to three-fold greater risk of getting MS than women in a subsequent stage, after not having it in the first stage. However, there was no difference between men and women in terms of the likelihood of recovery from MS, if they had it during the first stage. Aging increased the MS risk, and it also reduced the probability of recovery from MS. However, there was no advantage in terms of preventing health issues or improving health by reducing BMI when one had been overweight or obese in a previous stage. Gaining further education or an occupational promotion could independently reduce the probability of getting MS by 35% to 55%, compared to having a continuously low education status or unemployment; however, it could not improve the progression of MS for those who had the MS initially.

An adverse impact of continuing to smoke could be observed in subjects who suffered from MS, in that it reduced their probability of recovery by 42%, whereas prolonged drinking could increase the occurrence of MS by 70% compared to not drinking. Maintaining physical activity was advantageous in terms of the prevention and improvement of MS, but it did not have such effects if it was begun after the initial stage. A prolonged vegetarian diet or continued vitamin C/E intake could increase the probability of recovery from MS by 1.8 times and 68%, respectively, in comparison to the absence of these behaviors.

**Figure 1:**
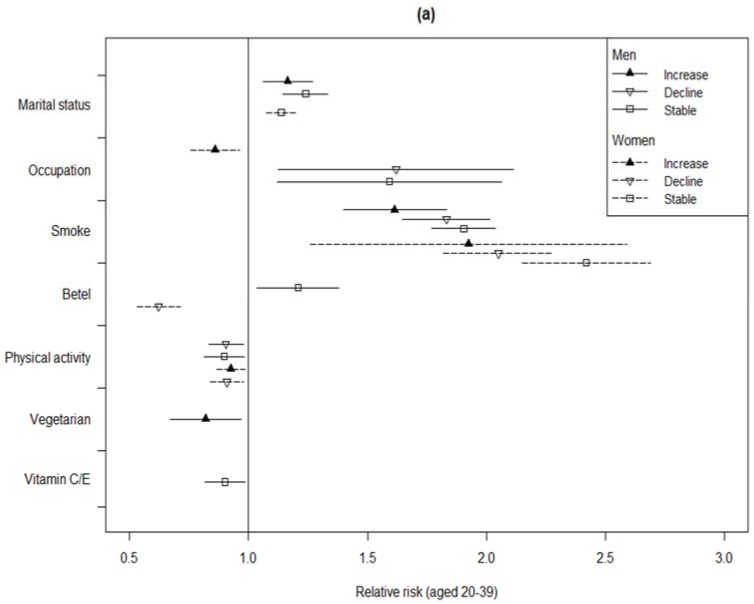

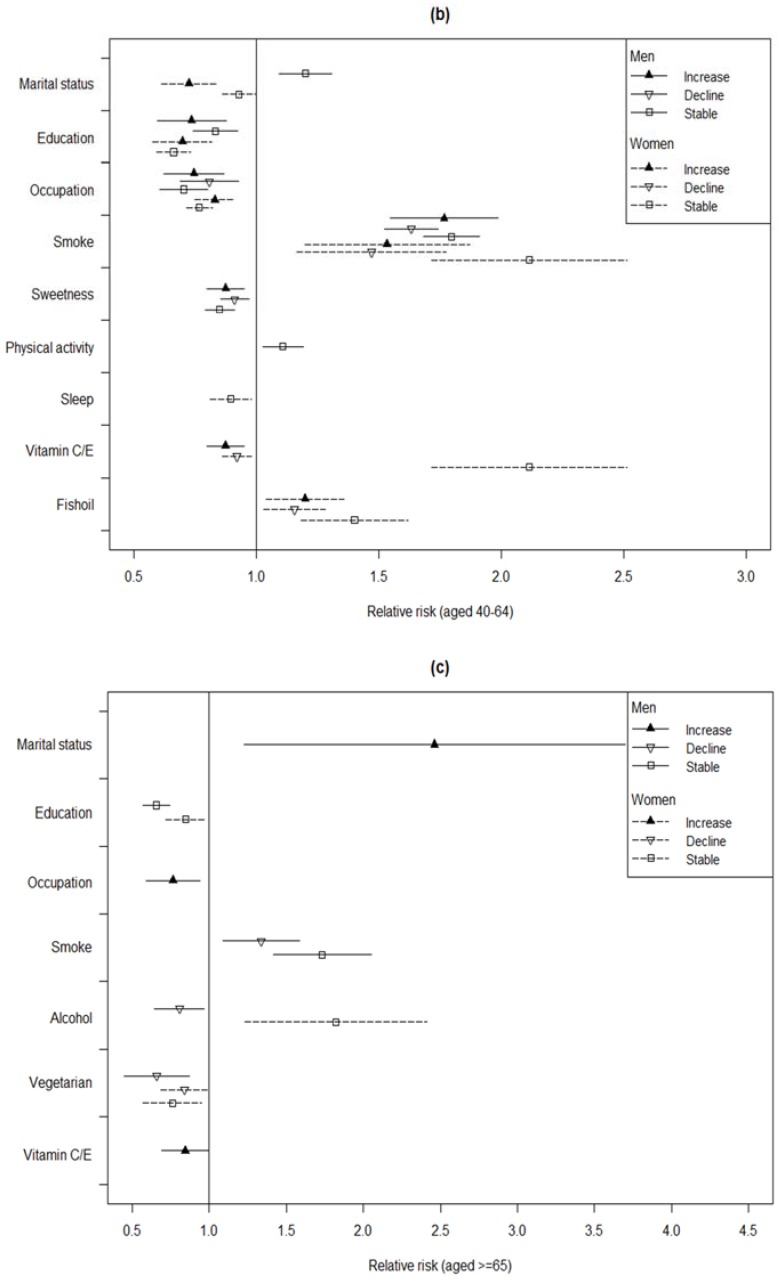
The relative risks (RRs) and 95% CIs of SES and lifestyle habits on 10-year Framingham risk score of cardiovascular disease (CVD) by sex- and age-stratified groups. Panel: (**a**) For subjects aged 20–39 years; (**b**) for subjects aged 40–64 years; (**c**) for aged ≥65 years. The statistics appropriate models were selected with Bayesian information criterion, based on the maximum likelihood method. Only RRs with significant values are shown, with these RRs being calculated by the GEE model adjusted with all covariates, including BMI and medicinal treatment. Each compared group (baseline) of explanatory variables was stable no/low status through the three study stages. The line presenting an increase, indicates the characteristic from absent to appear or the level from lower to higher, and vice versa.

The sex–age stratified impacts of changing SES and lifestyle habits on the 10-year estimated risk of CVD are shown in Figure 1. An estimated value, i.e., relative risk, above one for an explanatory variable indicated that it raised the risk of CVD independently compared to the baseline, with these estimated values being adjusted for medicinal treatment and BMI, as well as the other covariates. Unlike getting married or having a stable marriage, which could decrease the risk of CVD for middle-aged (40 to 64 years old) women by 7% to 28%, marriage was found to be a disadvantage that raised the CVD risk in men and young women. Meanwhile, gaining further education or an occupational promotion could reduce CVD risk for the majority of subjects. However, the risk of CVD was raised by half for young men (aged 20 to 40 years) who had a job or lost a job, compared to continuously unemployed young men. Smoking was found to be harmful for all populations, even if the habit had been ceased. Among the older subjects, a reduction in drinking could decrease the risk of CVD in men by 21%, whereas women who continued to increase their alcohol intake had about 72% of the risk of CVD, compared to women who did not drink at all. Continuous betel nut chewing was a hazard for young men, and ceasing the behavior was good for young women. Irrespective of gender, the advantage of physical activity on prevention was only observed in those aged lower than 40 years, whereas maintaining an exercise routine was useless for reducing CVD risk in middle-aged men. However, daily sweetened beverage drinking could decrease the CVD risk slightly for middle-aged men. Vegetarian diets were also helpful for reducing CVD risk for the older subjects by 17% to 27%, even if the habit was ceased in later stages. Irrespective of age, an advantage of vitamin C/E intake could be observed in men in particular. For middle-aged women, getting sufficient sleep could reduce CVD risk by 10%, but fish oil intake had the opposite effect.

## 4. Discussion

Our findings related to the effects of the factors on MS or CVD were consistent with those of past studies. The associations of MS or CVD with gender, age, and obesity have been well-explored [32,33]. The 10-year CVD risk predicted by FRS has been associated with social adversity during adulthood [34]. In addition to gaining education, the results of the current study indicated that occupation also played a positive role on health over the life course as well. Past studies have reported that socioeconomic adversities in various stages of life were associated with CVD incidence during adult life [35], as well as with a higher prevalence of risk factors for CVD [23,36]. Andrade et al. also suggested that low social class does not translate into a higher risk of CVD, among those who achieved a better SES in adulthood. The benefits of occupation to health may be linked to psychosocial stress [26]. Danese and McEwen suggested that chronic exposure to psychosocial stress through the life course triggers a subclinical atherosclerotic condition, which may promote CVD [37]. Therefore, we considered that getting a job or a promotion in occupation may increase the capability of an individual to improve his or her personal living and psychosocial conditions. Moreover, an individual’s awareness regarding quality of life will be raised with increasing educational attainment. Thus, both getting a job/promotion and increasing educational attainment could be advantageous for the prevention MS or CVD. Nonetheless, we found that these two factors did not help a person with MS recover to from it over several years. These results imply that good SES could be a protective factor against rather than a treatment for MS. On the other hand, we also found that having an occupation increases CVD risk in young men. This relationship could stem from young men finding it harder to endure the stress from work or other responsibilities than other populations. Further research could focus on taking stress measurements of young workers as they engage in work, to clarify this issue further. The advantage of stable marriage status also was found among the middle-aged women in our study. Karlamangla et al. found that the association between SES and cardiovascular risk accumulation was stronger in women than in men [25]. Relatedly, women who experienced a marital loss have a higher risk of CVD in late midlife, compared to continuously married women. Emotional distress and socioeconomic status account for the higher CVD risk among divorced women [38]. We suspect that as children reach adulthood and household-related workloads diminish, the stresses middle-aged married women have due to family concerns are alleviated, whilst also allowing these women to focus more on their own well-being. At the same time, family support can help these women feel better in terms of emotional satisfaction and safety, which may improve their psychological and physical health. In addition, the results of our study also indicated that getting sufficient sleep is also a protective factor for middle-aged women. Past studies have indicated that sleep disturbances can increase sympathetic activity, provoke systemic inflammation and oxidative stress, and impair vascular endothelial function. Moreover, short sleep durations and poor sleep quality have adverse effects on metabolic and hormonal processes, contributing to increased cardiovascular risk [39]. A recent European survey found that women’s sleep was more troubled by the presence of children in the home and having an unemployed partner, whereas men’s restless sleep was associated with their own unemployment and worries about household finances [40]. Those findings might explain our own findings that marriage can be a disadvantage in terms of CVD prevention in men, whilst being an advantage for middle-aged women. Taylor et al. suggested that women have a heightened biological sensitivity to SES [41]. Middle-aged women may gradually lose some of the protective effects they receive from estrogen as they pass through menopause, thus making them more susceptible to the effects of exposure to CVD risk factors in comparison to women in other life stages. Therefore, good social support and life conditions can play a more critical role in disease prevention in middle-aged women than in other populations.

The beneficial effects of physical activity in terms of the prevention and treatment of cardio-metabolic disorders are well-documented [42]. The current study further reveals that physical activity is helpful for the prevention of MS and CVD in young people. These results implied that exercise habits should be established relatively early, as the health disadvantages brought by insufficient exercise in early life cannot be diminished by increasing physical activity in later life. A British cohort study concluded that regular physical activity was associated with lower markers of inflammation over 10 years of follow-up, and that such activity may be important in preventing the pro-inflammatory state seen with ageing [43]. A recommendation from the American College of Sports Medicine and the American Heart Association was that all healthy adults aged 18 to 65 years engage in moderate-intensity aerobic (endurance) physical activity, for a minimum of 30 minutes on five days each week or vigorous-intensity aerobic PA for a minimum of 20 min on three days each week [44]. 

Past studies have reported the adverse effects of habits such as smoking, drinking, and betel nut chewing on health [32,45]. The present study finds that drinking primarily increases risk for MS or CVD in older individuals. Relatedly, decreased risks of MS and CVD can also be found in older men who quit drinking later in life. We considered that older people may be more vulnerable in general worsening health conditions. As such, quitting unhealthy habits may be more effective for them in terms of preventing them from getting CVD. In addition, the intake of supplements such as vitamin C/E or eating a vegetarian diet can also reduce the probability of CVD occurrence, especially in older individuals. These dietary habits have also been found to ease the suffering of those with MS. Relatedly, excessive oxidative stress has been most strongly implicated in chronic diseases such as CVD [46,47]. As such, the dietary intake of antioxidant agents, such as vitamin C, is associated with a lower risk of both MS and CVD [13]. Meanwhile, a Chinese study reported that vitamins C and E intake decreased with elevated numbers of MS components [14]. The current study found that the intake of those antioxidants in the diet or in nutritional supplements may be essential for health, particularly in men. The US recommended dietary vitamin C (vitamin E) intakes are 90 mg/day (15 mg/day) and 75 mg/day (15 mg/day) for male and female adults, respectively, and these levels of intake may effectively reduce the risk of MS [48]. The best sources of vitamin C are citrus fruit, dark green leafy vegetables, peppers, and dietary supplements. Natural vitamin E mainly comes from vegetable oils, unprocessed cereal grains, nuts, fruits, and vegetables [49]. Our findings also revealed that fish oil intake may not be advantageous in terms of preventing CVD, and that it may even, in fact, be harmful to health for middle-aged women. Further studies of the relevant etiological mechanism may be needed, but it is also possible that some of the study subjects misinterpreted the questions regarding fish oil intake included in the questionnaire.

The present study had several strengths. By using a large sample, it was able to explore more potential factors in its analysis, whilst also ensuring less variance, especially as it was a stratified study. The longitudinal design allowed us to simultaneously observe the changes in outcomes and their relevant factors over time, allowing them to be analyzed for their dynamic cause–effect associations, which have been less fully explored in past studies. Steele et al. suggested that the adoption of continuous risk scores seems to be plausible because such scores are statistically more sensitive and less susceptible to errors than dichotomous approaches [50]. By estimating two non-disease outcomes, i.e., dichotomous MS and continuous FSR, this study was also able to compare and elucidate the evidence associated with those outcomes, whilst exploring the early prevention strategies for sub-optimal health conditions. 

However, the approach of the current study had two key limitations. The first consisted of the effects of the selected institution itself. As the participants in this study were members of the medical facility who were apparently healthy adults, the prevalence of MS and FRS values among them may have been underestimated. Moreover, since the sample was not sampled randomly from the entire population of Taiwan, the results of the study should be interpreted with caution. Second, only the data for three examinations over nine years was collected and analyzed for each subject in the study, and even though data for each examination was collected, the status at the first examination is not truly representative of the initial status of each subject over his or her full life course. In other words, due to the lack of records from before the first examination, the study can only estimate the risk factors of intragenerational mobility at different life stages, rather than for the whole life. Intragenerational mobility is defined as the stability or change in SES over an individual’s life course, for example, from early adulthood to later adulthood [24,51]. 

Nonetheless, our findings do add some relevant knowledge regarding prevention. To reduce suffering due to MS or CVD, exercise habits should be developed relatively early in life and then maintained continuously. Furthermore, psychological training for stress control may be needed for young people as they engage in a job. In addition to avoiding obesity and unhealthy habits, maintaining stable family relationships, and getting sufficient sleep can diminish the risk of CVD for middle-aged women. Specific dietary habits such as taking vitamin C and E supplements and maintaining a vegetarian diet on a continuous basis are beneficial to health for men and older individuals, respectively.

## 5. Conclusions

The study presents evidence indicating that socioeconomic conditions and lifestyle behaviors may have different effects on health among various populations. The results elucidate the dynamic relationship between health outcomes and related factors over time more clearly, which may help people to take more efficient disease prevention measures. Related suggestions can be provided to healthcare workers as they seek to design health promotion courses for populations at different life stages. 

## Figures and Tables

**Table 1:** Sociodemographic characteristics and lifestyle habits related to metabolic syndrome (MS) over the three study stages.

Characteristics	MS in 2006–2008	MS in 2009–2011	MS in 2012–2014
	*n* (%)	*n* (%)	*n* (%)
Sex			
Male	2097 (31.70)	2555 (38.62)	2956 (44.68)
Female	799 (12.77)	1031 (16.60)	1327 (21.37)
Age (years)			
20–39	863 (13.14)	856 (16.84)	735 (19.77)
40–64	1741 (29.95)	2299 (32.32)	2966 (35.83)
≥65	292 (65.47)	431 (68.74)	582 (70.21)
Socioeconomic status			
Marital status			
Unmarried	572 (17.11)	639 (22.37)	723 (28.17)
Married	2324 (24.51)	2947 (29.56)	3560 (34.70)
Education (years)			
<12	448 (52.03)	513 (59.51)	570 (65.74)
12–15	1135 (21.76)	1429 (28.16)	1666 (33.49)
≥16	1313 (19.45)	1644 (23.87)	2047 (29.31)
Income (NTD/year)	378 (26.81)	434 (33.80)	550 (42.80)
<400,000	649 (19.42)	785 (26.45)	828 (30.53)
400,000–800,000	1869 (23.15)	2367 (27.61)	2905 (32.91)
>800,000			
Occupation			
Unemployment	634 (29.71)	787 (35.24)	999 (42.37)
Managed	443 (28.00)	550 (32.62)	696 (39.08)
Non-managed	1819 (19.97)	2249 (25.25)	2588 (29.80)
Life style			
Smoke (pack /week)			
None	2353 (21.32)	2977 (26.41)	3640 (31.89)
1–6	421 (28.47)	475 (36.43)	499 (43.26)
≥7	122 (39.61)	134 (53.82)	144 (56.69)
Alcohol (cup/week)			
None	2332 (20.84)	2932 (26.17)	3504 (31.46)
1–6	260 (29.78)	323 (37.65)	387 (42.02)
≥7	304 (39.95)	331 (43.27)	392 (51.17)
Sweetened beverage (cup/week)			
None	981 (25.00)	1713 (31.21)	2249 (35.33)
1–6	1370 (22.24)	1420 (26.20)	1549 (31.64)
≥7	545 (19.89)	453 (23.63)	485 (31.03)
Chewing betel nut			
No	2699 (21.87)	3359 (27.10)	4050 (32.66)
Yes	197 (40.87)	227 (52.91)	233 (55.08)
Physical activity (h/week)			
<1	961 (18.63)	1427 (24.49)	1653 (31.02)
1–6	1757 (24.33)	1810 (29.99)	2226 (34.14)
≥7	178 (39.91)	349 (36.28)	404 (41.39)
Sleep (h/day)			
<6	637 (25.24)	892 (31.11)	1210 (37.51)
6	2075 (21.89)	1862 (28.38)	2117 (32.57)
≥7	184 (22.36)	832 (24.48)	956 (30.85)
Vegetarian diet			
Yes	61 (18.77)	70 (18.82)	97 (23.15)
No	2835 (22.68)	3516 (28.23)	4186 (33.74)
Vitamin C, E intake			
Yes	474 (19.94)	496 (23.85)	577 (29.85)
No	2422 (23.18)	3090 (28.76)	3706 (34.02)
Fish oil			
Yes	304 (33.85)	369 (36.79)	527 (39.56)
No	2592 (21.73)	3217 (27.21)	3756 (32.68)

**Table 2:** Risk indicators of metabolic syndrome and cardiovascular diseases over the three study stages.

Indicators	MS in 2006–2008	MS in 2009–2011	MS in 2012–2014
	*n* (%)	*n* (%)	*n* (%)
BMI			
<24	942 (11.14)	1145 (14.13)	1359 (17.64)
24–26.9	978 (33.53)	1280 (41.78)	1483 (45.84)
≥27	976 (67.17)	1161 (70.02)	1441 (76.49)
Waist (cm)			
<80(F); <90(M)	1582 (14.37)	1989 (18.41)	2300 (22.08)
Otherwise	1314 (72.24)	1597 (79.06)	1983 (82.28)
Fasting glucose (mg/dL)			
<100	657 (7.69)	743 (9.68)	768 (11.34)
≥100	2239 (52.24)	2843 (55.24)	3515 (58.10)
Triglyceride (mg/dL)			
<150	1475 (13.98)	1836 (17.73)	2305 (22.62)
≥150	1421 (62.52)	1750 (70.91)	1978 (75.09)
HDL (mg/dL)			
≥40 (M); ≥50(F)	2367 (22.47)	3213 (27.85)	3807 (33.22)
Otherwise	529 (23.09)	373 (28.98)	476 (34.87)
LDL (mg/dL)			
<130	1771 (19.19)	2372 (24.67)	2359 (27.98)
≥130	1125 (31.28)	1214 (37.81)	1924 (43.79)
TC (mg/dL)			
<200	1340 (16.85)	1650 (21.56)	1960 (27.15)
200–239	1156 (29.44)	1463 (35.13)	1775 (40.10)
≥240	400 (42.24)	473 (47.02)	548 (46.44)
Hypertension			
Normal	1088 (10.59)	1537 (15.09)	1911 (19.47)
High normal	975 (63.93)	1109 (72.82)	1210 (74.46)
Phase 1	679 (80.26)	779 (83.76)	940 (82.89)
Phase 2–3	147 (84.48)	153 (86.44)	217 (88.21)
Other	7 (77.78)	8 (88.89)	5 (71.43)
Medicine			
Antihyperglycemic drug			
Yes	188 (100.00)	282 (100.00)	372 (100.00)
No	2708 (21.43)	3304 (26.34)	3911 (31.41)
Antihyperlipidemic drug			
Yes	184 (100.00)	300 (100.00)	410 (100.00)
No	2712 (21.45)	3286 (26.24)	3873 (31.20)
Antihypertensive drug			
Yes	733 (100.00)	995 (100.00)	1237 (100.00)
No	2163 (17.89)	2591 (21.90)	3046 (26.29)
Framingham CVD score			
Mean (standard deviation)	0.014 (0.015)	0.014 (0.014)	0.015 (0.016)
Medium	0.009	0.009	0.010

**Table 3:** Odds ratios (95% confidence intervals) of socioeconomic patterns and lifestyle conditions for metabolic syndrome over the three study stages.

Heading	Without MS in 2006–2008 (n = 9929)				MS in 2006–2008 (n = 2896)			
	Worse (NNP/NPP vs NNN)		Unstable (NPN vs NNN)		Improve (PNN/PPN vs PPP)		Unstable (PNP vs PPP)	
	AOR	95% CI	AOR	95% CI	AOR	95% CI	AOR	95% CI
Male (vs female)	2.30	(1.99, 2.65)	3.01	(2.32, 3.89)	1.05	(0.71, 1.55)	0.88	(0.63, 1.21)
Age in 2006–2008 (vs 20–39)								
40–64	1.67	(1.44, 1.95)	1.55	(1.17, 2.05)	0.51	(0.36, 0.72)	0.80	(0.52, 1.20)
≥65	2.25	(1.50, 3.28)	3.30	(1.80, 6.02)	0.47	(0.26, 0.86)	0.42	(0.21, 0.87)
BMI (vs Normal)								
Increase	5.51	(4.71, 6.44)	2.69	(1.98, 3.66)	0.19	(0.12, 0.30)	1.01	(0.66, 1.56)
Decline	1.09	(0.74, 1.59)	1.62	(0.96, 2.71)	1.44	(0.98, 2.11)	1.00	(0.54, 1.68)
Stable overweight/obesity	6.24	(5.36, 7.28)	4.03	(3.10, 5.25)	0.23	(0.17, 0.31)	0.54	(0.38, 0.77)
Unstable condition	3.39	(2.53, 4.55)	5.49	(3.68, 8.16)	0.51	(0.31, 0.85)	0.74	(0.38, 1.42)
Socioeconomics								
Marital status (vs unmarried)								
Get married	1.02	(0.79, 1.31)	1.03	(0.66, 1.60)	0.62	(0.34, 1.13)	0.93	(0.48, 1.79)
Divorced/widowed	1.06	(0.66, 1.70)	1.39	(0.67, 2.90)	0.71	(0.29, 1.71)	0.55	(0.18, 1.70)
Married	0.97	(0.81, 1.16)	0.92	(0.67, 1.25)	0.87	(0.61, 1.22)	0.88	(0.58, 1.32)
Unstable condition	1.08	(0.41, 2.82)	4.11	(1.44, 11.68)	0.77	(0.58, 1.32)	<0.001	-
Education (vs <12 years)								
Increase	0.45	(0.28, 0.73)	0.60	(0.26, 1.40)	1.46	(0.58, 3.66)	2.18	(0.81, 5.89)
Stable medium/high education	0.48	(0.35, 0.66)	0.61	(0.35, 1.07)	1.17	(0.70, 1.94)	1.31	(0.73, 2.33)
Others	0.56	(0.30, 1.02)	0.62	(0.21, 1.81)	0.88	(0.29, 2.69)	0.89	(0.23, 3.43)
Income (vs <400,000 NTD)								
Increase	1.11	(0.78, 1.59)	1.43	(0.76,2.72)	0.86	(0.44, 1.67)	0.67	(0.39, 1.31)
Decline	1.16	(0.79, 1.68)	1.40	(0.72,2.73)	1.24	(0.64, 2.41)	0.54	(0.26, 1.15)
Stable medium/high income	1.07	(0.76, 1.50)	1.17	(0.63,2.18)	0.94	(0.51, 1.74)	0.46	(0.24, 0.87)
Unstable condition	0.89	(0.61, 1.31)	0.94	(0.46,1.89)	1.54	(0.79, 3.02)	0.89	(0.44, 1.81)
Occupation (vs unemployed)								
Promotion	0.65	(0.47, 0.89)	0.75	(0.43, 1.31)	1.76	(0.93, 3.33)	1.03	(0.51, 2.06)
Demotion	0.90	(0.67, 1.17)	0.60	(0.35, 1.03)	1.35	(0.78, 2.36)	0.60	(0.43, 1.31)
Stable position	0.68	(0.54, 0.86)	0.85	(0.56, 1.29)	1.18	(0.72, 1.93)	0.64	(0.38, 1.08)
Unstable condition	0.71	(0.51, 1.00)	0.93	(0.53, 1.64)	1.29	(0.64, 2.59)	0.65	(0.29, 1.45)
Lifestyle								
Smoking (vs none)								
Increase	0.64	(0.39, 1.04)	0.60	(0.23, 1.55)	1.74	(0.85, 3.54)	0.38	(0.09, 1.65)
Decline	1.10	(0.83, 1.45)	1.26	(0.80, 1.97)	1.17	(0.71, 1.94)	1.24	(0.69, 2.22)
Stable intake	1.19	(0.95, 1.47)	1.30	(0.91, 1.87)	0.58	(0.38, 0.89)	0.89	(0.56, 1.40)
Unstable condition	0.89	(0.54, 1.49)	1.00	(0.41, 2.38)	0.40	(0.15, 1.06)	0.41	(0.12, 1.46)
Alcohol intake (vs none)								
Increase	1.17	(0.92, 1.49)	0.99	(0.66, 1.50)	0.92	(0.58, 1.48)	0.99	(0.56, 1.75)
Decline	1.00	(0.76, 1.29)	0.73	(0.45, 1.18)	1.33	(0.86, 2.06)	0.99	(0.56, 1.75)
Stable intake	1.69	(1.22, 2.33)	0.67	(0.33, 1.37)	0.58	(0.33, 1.01)	0.76	(0.39, 1.46)
Unstable condition	0.96	(0.72, 1.29)	0.74	(0.44, 1.26)	0.83	(0.50, 1.37)	1.35	(0.80, 2.27)
Sweetened beverage intake (vs none)								
Increase	1.20	(0.94, 1.52)	1.13	(0.76, 1.69)	1.17	(0.75, 1.88)	1.57	(0.92, 2.67)
Decline	1.11	(0.92, 1.34)	1.08	(0.79, 1.48)	0.97	(0.69, 1.37)	0.98	(0.64, 1.51)
Stable intake	1.17	(0.96, 1.44)	0.99	(0.70, 1.42)	1.21	(0.82, 1.77)	1.33	(0.84, 2.12)
Unstable condition	1.17	(0.95, 1.45)	1.00	(0.69, 1.45)	1.00	(0.66, 1.48)	1.23	(0.76, 1.99)
Betel nut chewing (vs none)								
Increase	1.19	(0.63, 2.28)	1.07	(0.36, 3.18)	1.25	(0.37, 4.16)	0.42	(0.05, 3.45)
Decline	1.34	(0.76, 2.33)	0.58	(0.17, 1.96)	0.90	(0.35, 2.34)	1.23	(0.50, 3.04)
Stable intake	1.40	(0.94, 2.08)	1.44	(0.75, 2.75)	1.45	(0.78, 2.70)	0.78	(0.35, 1.78)
Unstable condition	1.38	(0.73, 2.60)	0.86	(0.25, 2.94)	0.55	(0.15, 2.05)	0.27	(0.04, 2.15)
Physical activity (vs none)								
Increase	0.89	(0.73, 1.08)	0.99	(0.70, 1.40)	1.43	(0.95, 2.16)	0.92	(0.57, 1.48)
Decline	0.95	(0.78, 1.15)	0.90	(0.63, 1.28)	1.74	(1.16, 2.60)	1.15	(0.73, 1.81)
Stable medium/high	0.80	(0.66, 0.97)	1.06	(0.77, 1.47)	1.54	(1.04, 2.27)	1.03	(0.66, 1.60)
Unstable condition	0.85	(0.70, 1.04)	0.91	(0.64, 1.29)	1.05	(0.69, 1.60)	0.81	(0.50, 1.30)
Sleep (vs <6 h)								
Increase	1.02	(0.81, 1.29)	1.53	(0.99, 2.37)	1.12	(0.72, 1.75)	1.27	(0.74, 2.18)
Decline	0.91	(0.70, 1.17)	1.37	(0.85, 2.21)	0.97	(0.60, 1.57)	1.20	(0.67, 2.13)
Stable medium/high	0.94	(0.75, 1.181	1.18	(0.77, 1.83)	1.18	(0.77, 1.83)	1.38	(0.82, 2.34)
Unstable condition	0.88	(0.69, 1.13)	1.03	(0.65, 1.65)	0.92	(0.57, 1.48)	1.36	(0.77, 2.39)
Vegetarian diet (vs not)								
Increase	1.12	(0.67, 1.88)	1.19	(0.51, 2.81)	2.39	(0.78, 7.31)	1.69	(0.42, 6.80)
Decline	0.63	(0.29, 1.36)	1.19	(0.40, 3.53)	0.27	(0.03, 2.36)	0.79	(0.15, 4.10)
Stable intake	0.61	(0.37, 1.01)	0.43	(0.15, 1.20)	2.81	(1.21, 6.55)	3.03	(1.19, 7.75)
Unstable condition	0.84	(0.35, 2.01)	0.49	(0.07, 3.67)	<0.001	-	0.78	(0.09, 6.56)
Vitamin C, E intake (vs none)								
Increase	0.97	(0.78, 1.21)	0.95	(0.65, 1.40)	1.26	(0.84, 1.88)	1.67	(1.05, 2.67)
Decline	0.84	(0.69, 1.06)	0.72	(0.50, 1.04)	1.20	(0.83, 1.74)	1.32	(0.85, 2.03)
Stable intake	0.97	(0.79, 1.19)	1.18	(0.84, 1.65)	1.68	(1.12, 2.52)	1.42	(0.87, 2.31)
Unstable condition	0.81	(0.39, 1.23)	1.71	(0.98, 3.01)	1.37	(0.71, 2.67)	1.11	(0.45, 2.78
Fish oil intake (vs none)								
Increase	1.10	(0.87, 1.39)	1.07	(0.72, 1.58)	0.76	(0.49, 1.17)	0.73	(0.43, 1.25)
Decline	1.34	(0.99, 1.81)	0.96	(0.55, 1.65)	0.67	(0.37, 1.17)	0.84	(0.44, 1.60)
Stable intake	1.10	(0.67, 1.79)	0.98	(0.41, 2.34)	1.05	(0.43, 1.25)	0.51	(0.18, 1.47)
Unstable condition	0.93	(0.66, 1.31)	0.83	(0.45, 1.52)	0.84	(0.48, 1.47)	0.85	(0.43, 1.68)

P: positive; N: negative; three letters present the status across the three stages. For example, NPP is defined as the occurrence of MS in the second and third stage for those without MS in the first stage, and vice versa. Adjusted odds ratios (AOR) are calculated by the logistic regression model adjusted with all covariates, including medicinal treatment. Each compared group (baseline) of explanation variables is stable, no, or low status through three study stages.
